# Familial Clusters of HTLV-1-Associated Myelopathy/Tropical Spastic Paraparesis

**DOI:** 10.1371/journal.pone.0086144

**Published:** 2014-05-06

**Authors:** Satoshi Nozuma, Eiji Matsuura, Toshio Matsuzaki, Osamu Watanabe, Ryuji Kubota, Shuji Izumo, Hiroshi Takashima

**Affiliations:** 1 Department of Neurology and Geriatrics, Kagoshima University Graduate School of Medical and Dental Sciences, Kagoshima city, Japan; 2 Department of Molecular Pathology, Center for Chronic Viral Diseases, Kagoshima University Graduate School of Medical and Dental Sciences, Kagoshima city, Japan; National Institutes of Health, United States of America

## Abstract

**Objective:**

HTLV-1 proviral loads (PVLs) and some genetic factors are reported to be associated with the development of HTLV-1-associated myelopathy/tropical spastic paraparesis (HAM/TSP). However, there are very few reports on HAM/TSP having family history. We aimed to define the clinical features and laboratory indications associated with HAM/TSP having family history.

**Methods:**

Records of 784 HAM/TSP patients who were hospitalized in Kagoshima University Hospital and related hospitals from 1987 to 2012 were reviewed. Using an unmatched case-control design, 40 patients of HAM/TSP having family history (f-HAM/TSP) were compared with 124 patients suffering from sporadic HAM/TSP, who were admitted in series over the last 10 years for associated clinical features.

**Results:**

Of the 784 patients, 40 (5.1%) were f-HAM/TSP cases. Compared with sporadic cases, the age of onset was earlier (41.3 vs. 51.6 years, p<0.001), motor disability grades were lower (4.0 vs. 4.9, p = 0.043) despite longer duration of illness (14.3 vs. 10.2 years, p = 0.026), time elapsed between onset and wheelchair use in daily life was longer (18.3 vs. 10.0 years, p = 0.025), cases with rapid disease progression were fewer (10.0% vs. 28.2%, p = 0.019), and protein levels in cerebrospinal fluid (CSF) were significantly lower in f-HAM/TSP cases (29.9 vs. 42.5 mg, p<0.001). There was no difference in HTLV-1 PVLs, anti-HTLV-1 antibody titers in serum and CSF, or cell number and neopterin levels in CSF. Furthermore, HTLV-1 PVLs were lower in cases with rapid disease progression than in those with slow progression in both f-HAM/TSP and sporadic cases.

**Conclusions:**

We demonstrated that HAM/TSP aggregates in the family, with a younger age of onset and a slow rate of progression in f-HAM/TSP cases compared with sporadic cases. These data also suggested that factors other than HTLV-1 PVLs contribute to the disease course of HAM/TSP.

## Introduction

HTLV-1-associated myelopathy/tropical spastic paraparesis (HAM/TSP) is characterized by slow progressive spastic paraparesis and positivity for anti-HTLV-1 antibodies in both serum and cerebrospinal fluid (CSF) [1], [2]. Worldwide, at least 10–20 million people are infected with HTLV-1 [3]. However, although the majority of infected individuals remain lifelong asymptomatic carriers, approximately 2%–5% develop adult T-cell lymphomas [4], [5] and another 0.25%–3.8% develop HAM/TSP [1], [2]. Although the mechanisms underlying the development of HAM/TSP are not fully understood, several risk factors are closely associated with HAM/TSP. In particular, HTLV-1 proviral loads (PVLs) are significantly higher in HAM/TSP patients than in asymptomatic carriers and are also higher in genetic relatives of HAM/TSP patients than in non-HAM-related asymptomatic carriers [6]. Host genetic factors, including human leukocyte antigen (HLA) and non-HLA gene polymorphisms affect the occurrence of HAM/TSP [7], indicating that HTLV-1 PVLs and genetic backgrounds may influence individual susceptibility to HAM/TSP. Although several reports of familial adult T-cell lymphoma have been published [8], [9], to our knowledge, there is only one case report of patient with HAM/TSP having family history (f-HAM/TSP) [10]. Hence, little is known about the prevalence and character of f-HAM/TSP cases. In this study, the characteristic clinical and laboratory features of f-HAM/TSP cases are defined and compared with those of sporadic cases.

## Methods

### Ethics Statement

This study was approved by the Institutional Review Boards of Kagoshima University. All participants provided written informed consent.

### Design

We used an unmatched case-control design to identify the phenotypic features of f-HAM/TSP. f-HAM/TSP cases were identified as patients with multiple family members suffering from HAM/TSP. Controls were defined as HAM/TSP patients who were not genetically related to other HAM/TSP patients.

### Subjects

f-HAM/TSP cases were extracted from our database of individuals diagnosed with HAM/TSP in Kagoshima University Hospital and related hospitals from 1987 to 2012. Controls included consecutive patients with sporadic HAM/TSP who were evaluated in our department between January 2002 and June 2012. HAM/TSP was diagnosed according to the World Health Organization diagnostic criteria, and the updated criteria of Castro-costa Belem [11]. Clinical information was obtained from the medical records of patient attendance at our hospital. In other cases, clinical data were obtained from the clinical records of patients or directly from the referring clinicians. Clinical variables included sex, age, age of onset, and initial symptoms. Neurological disabilities were assessed using Motor Disability Grading (MDG), modified from the Osame Motor Disability Scale of 0 to 10, as reported previously [12]. Motor disability grades were defined as follows: 5, needs one-hand support while walking; 6, needs two-hand support while walking; and 7, unable to walk but can crawl. We used a different assessment for the subgroup of more than grade 6 because their disease state significantly interfered with their lifestyle and necessitated the use of wheelchairs in daily life. The subgroup of patients with rapid progression was defined by deterioration of motor disability by more than three grades within two years. Anti-HTLV-1 antibody titers in serum and CSF were detected using enzyme-linked immunosorbent assays and particle agglutination methods (Fijirebio Inc, Tokyo, Japan). HTLV-1 PVLs in peripheral blood mononuclear cells (PBMCs) were assayed using quantitative PCR with the ABI PRISM 7700TM sequence detection system as reported previously [6].

## Statistical Analysis

Data were analyzed using SPSS-20 (SPSS, Chicago, Illinois). Statistical analyses were performed using parametric (t-test) and non-parametric tests (Mann–Whitney test) for continuous variables and χ^2^ (Pearsonχ^2^ test/Fisher exact test) for categorical variables. Significant differences were then adjusted for potential confounders (age and sex) using multiple linear regression analysis. Survival was estimated according to the Kaplan–Meier method. The final endpoint was defined by a MDG score of 6. Patients with MDG scores of 6 almost wheelchair bound in daily life. The log rank test was used in Kaplan–Meier analyses. Differences were considered significant when p<0.05.

## Results

### Clinical characteristics of f-HAM/TSP

**Figure 1:**
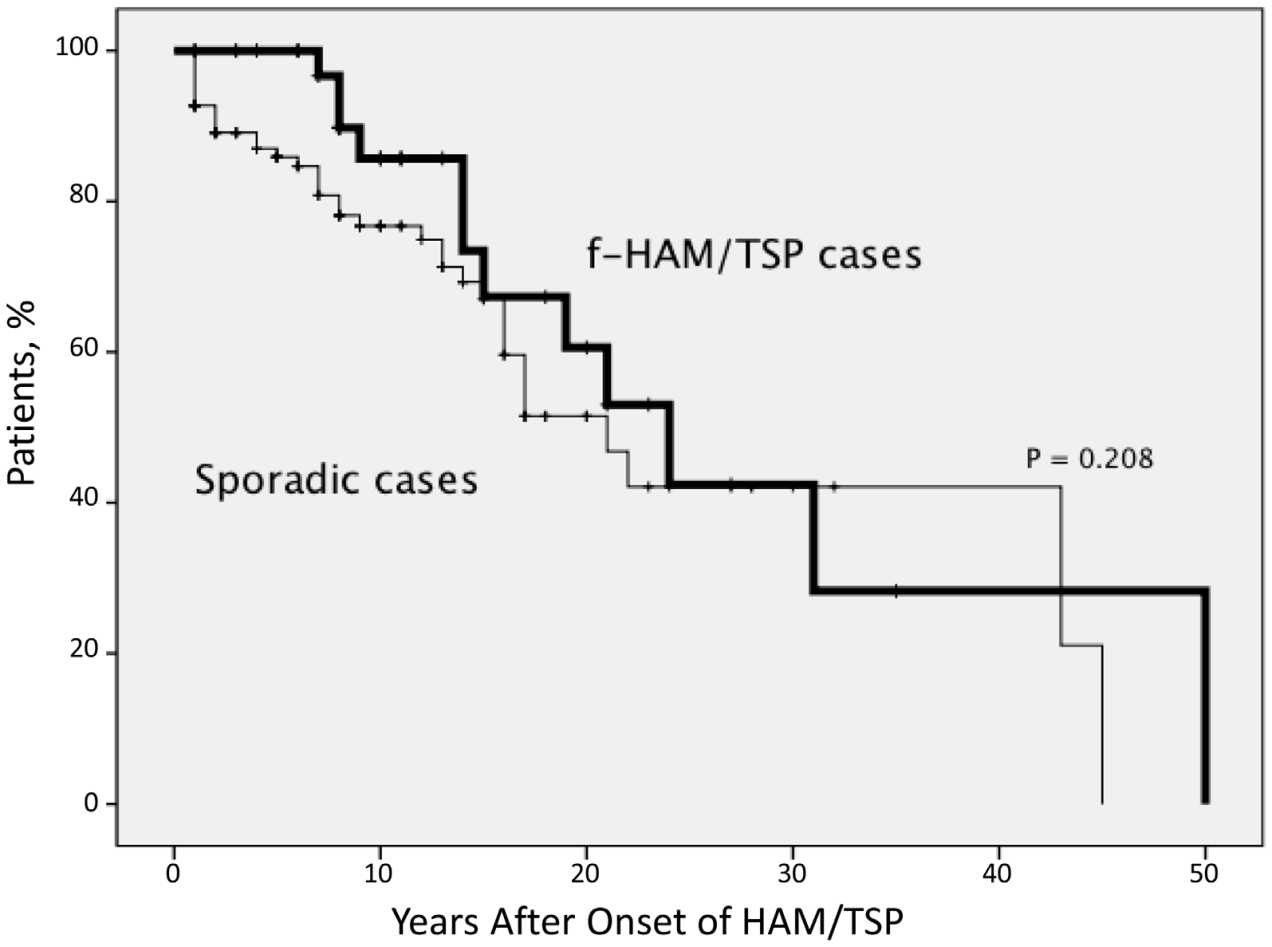
Kaplan–Meier estimates of the time from disease onset to assignment of motor disability scores of 6. In sporadic cases, more patients reached the score of six at an early stage; however, the difference was not significant. Approximately 30% of both f-HAM/TSP cases and sporadic cases needed a wheelchair in daily life in 15 years after onset and approximately 50% of patients from both groups needed a wheelchair in 20 years after onset.

Of the 784 patients diagnosed with HAM/TSP between January 1987 and June 2012, 40 (5.1%) were f-HAM/TSP. The sex ratio was 33 males : 7 females. Of these 40 cases, 10 had parents or children (25.0%), 27 had siblings (67.5%), and three had other relatives (7.5%) diagnosed with HAM/TSP. Three individuals from one family were diagnosed with HAM/TSP, whereas only two individuals were diagnosed with HAM/TSP in all other families. In f-HAM/TSP cases, the age of onset was earlier (41.3 vs. 51.6 years, p<0.001), cases with rapid progression were fewer (10.0% vs. 28.2%, p = 0.019), motor disability grades were lower (4.0 vs. 4.9, p = 0.043) despite longer duration of illness (14.3 vs. 10.2 years, p = 0.026), and time elapsed between onset and wheelchair use in daily life was longer (18.3 vs. 10.0 years, p = 0.025) compared with sporadic cases. Sex and initial symptoms did not differ significantly between f-HAM/TSP and sporadic cases (Table 1). Twelve patients of f-HAM/TSP, and 38 of the 128 sporadic cases reached endpoint MDG scores of 6. Significant differences were then adjusted for potential confounders (age and sex) using multivariate analysis. Age of onset, duration of illness, MDG scores, and time elapsed between onset and wheelchair use in daily life remained significantly different after multivariate analysis (Table 1). The proportion of patients with rapid progression did not differ significantly between the groups, although there was a trend toward a higher proportion in sporadic cases. Kaplan–Meier analyses revealed that approximately 30% of both f-HAM/TSP and sporadic cases needed a wheelchair in daily life in 15 years after onset, and approximately 50% of patients from both groups needed it in 20 years after onset (Figure 1). Although sporadic patients needed wheelchairs earlier in most cases, the difference in the ratio of the patients with MDG score above six was not statistically significant between the groups. Finally, we compared differences in the age of onset between parent–child and sibling cases in f-HAM/TSP cases. Age of onset in parent–child f-HAM/TSP cases was significantly younger than that in sibling f-HAM/TSP cases (29.9±10.0 vs. 45.1±13.0 years, p = 0.002).

**Table 1:** Clinical features of f-HAM/TSP cases or sporadic cases of HAM/TSP.

	f-HAM/TSP cases (40 cases)	Sporadic cases (124 cases)	p value	p value†
Female ratio (%)	78.8% (7 males : 33 females)	66.4% (31 males : 93 females)	NS	
Age	55.6±13.0 (23–79)	61.8±12.5 (15–83)	**0.008**	
Age of onset	41.3±13.9 (14–65)	51.6±15.9 (13–78)	**<0.001**	**0.017**
Duration of illness (years)	14.3±11.4 (1–49)	10.2±9.6 (0–45)	**0.026**	**0.017**
Initial symptoms				
Gait disturbance	50.0%	52.4%	NS	
Urinary disturbance	32.5%	26.6%	NS	
Sensory disturbance	12.5%	14.5%	NS	
Others	5%	6.5%	NS	
Rapid disease progression	4 cases (10.0%)	35 cases (28.2%)	**0.019**	0.069
Motor disability score	4.0±2.0 (0–7)	4.9±1.5 (0–8)	**0.043**	**0.036**
Score more than 6	12 cases (30.0%)	38 cases (30.7%)	NS	
Time elapsed between onset and wheelchair use in daily life (years)	18.3±12.4 (7–50)	10.0±10.4 (1–45)	**0.025**	**0.020**

Data are presented as mean values ± s.d., (range),

† Adjusted for age and sex.

### Laboratory parameters and PVLs in f-HAM/TSP cases

Protein levels in CSF were significantly lower in f-HAM/TSP cases than in sporadic cases (29.9 vs. 42.5 mg/dl, p<0.001). This difference in CSF protein level remained significant after multivariate analysis. Anti-HTLV-1 antibody titers in serum and CSF, and cell numbers and neopterin levels in CSF were not significantly different between two groups. Moreover, HTLV-1 PVLs did not differ significantly. (Table 2).

**Table 2:** Laboratory findings of familial clusters or sporadic cases of HAM/TSP.

	f-HAM/TSP cases (40cases)	Sporadic cases (124 cases)	p value	p value†
Anti-HTLV-1 antibodies*				
Titer in Serum	20,787±31,004, N = 37	31,009±36,075, N = 109	NS	
Titer in CSF	2,310±11,741, N = 31	672±1,274, N = 111	NS	
Cerebrospinal fluid				
Cell number (/mm^3^)	3.0±2.5, N = 25	5.7±10.0, N = 109	NS	
Protein (mg/dl)	29.9±9.4, N = 22	42.5±19.3, N = 109	**<0.001**	**0.007**
Neopterin (pmol/ml)	83.2±118.1, N = 18	38.3±56.8, N = 35	NS	
HTLV-1 proviral loads (Copies/10^4^ PBMCs)	930±781, N = 32	968±1,746, N = 101	NS	

* Particle Aggregation Method.

Data are presented as mean values ± s.d., N = sample number,

† Adjusted for age and sex.

### Clinical and laboratory findings in patients with rapid disease progression

**Figure 2:**
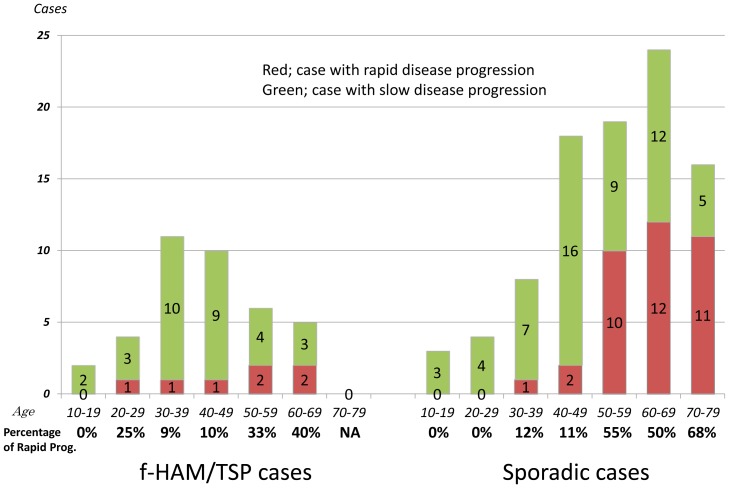
Age-specific proportions of rapid disease progression. The proportion of cases with rapid disease progression tended to increase with the older age of onset.

Previous studies suggest that an older age of onset is associated with rapid disease progression. Similar findings are found in the present study. The percentage of rapid progression tended to increase with older age of onset in both f-HAM/TSP and sporadic groups (Figure 2). We compared the characteristics of 124 sporadic HAM/TSP patients with rapid and slow progression who were admitted to Kagoshima University Hospital in series during the last 10 years (Table 3). Patients with rapid progression were significantly older at onset than those with slow progression (62.3 vs. 47.4 years, p<0.001), although sex and initial symptoms did not differ significantly between rapid and slow progression groups. However, the time elapsed between onset and wheelchair use in daily life was markedly shorter among patients with rapid progression (1.5 vs. 14.4 years, p<0.001). Cell numbers, protein levels, and anti-HTLV-1 antibody titers in CSF were significantly higher in patients with rapid progression than in those with slow progression (11.6 vs. 3.2, p<0.001; 55.3 vs. 36.7 mg/dl, p<0.001; 1,251 vs. 416, p<0.014, respectively). Interestingly, HTLV-1 PVLs were significantly lower in patients with rapid progression than in those with slow progression (370 vs. 1,245 copies, p<0.001). Furthermore, we compared the differences between women and men in patients with rapid progression because the reason remains unknown why HAM/TSP is common in female than in male. There was no significant difference between women and men in the age of onset (61.5 y.o.±12.6 vs. 62.7 y.o.±12.5), in the incidence of rapid progression (26.3% vs. 32.3%) and in MDG score (5.4 vs. 5.0; mean).

**Table 3:** Clinical and laboratory findings of sporadic HAM/TSP with rapid/slow disease progression.

Type of disease progression	Rapid progression	Slow progression	p value
Female ratio (%)	71.4% (10 males : 25 females)	76.4% (21 males : 68 females)	NS
Age of onset	62.3±9.6, N = 35	47.4±15.9, N = 89	**<0.001**
Age of onset of f-HAM/TSP cases	60.5±3.7, N = 4	39.2±12.9, N = 36	**0.002**
Duration between onset and inability to walk alone (years)	1.5±0.9, N = 13	14.4±10.4, N = 25	**<0.001**
Anti-HTLV-1 antibodies*			
Titer in Serum	31,894±36,845, N = 34	30,608±35,965, N = 75	NS
Titer in CSF	1,251±1,800, N = 34	416±852, N = 77	**0.014**
Cerebrospinal fluid			
Cell number (/mm^3^)	11.6±16.6, N = 34	3.2±3.5, N = 75	**<0.001**
Protein (mg/dl)	55.3±24.3, N = 34	36.7±13.0, N = 75	**<0.001**
Neopterin (pmol/ml)	74.9±107.9, N = 8	27.4±23.4, N = 27	0.255
HTLV-1 proviral loads (Copies/10^4^ PBMCs)	370±327, N = 32	1,245±2,046, N = 69	**<0.001**

* Particle Aggregation Method.

Data are presented as mean values ± s.d., N = sample number.

## Discussion

We demonstrated that among 784 HAM/TSP patients, 40 (5.1%) had family members with the disease. The lifetime risk of developing HAM/TSP is 0.25% of HTLV-1 carriers in Japan [13]. Although clustering of familial adult T-cell lymphomas has been reported [8], [9], to our knowledge the prevalence of familial clusters of HAM/TSP has not been described. A study in Peru showed that 30% of HAM/TSP patients have family members with paralytic neurological disorders, but the cause of paralysis was not evaluated [14]. In the present study, we included f-HAM/TSP diagnosed in medical institutions and excluded cases with a family history of neurological disorders. Thus, the actual incidence rates of f-HAM/TSP may be higher than those reported here. Interestingly, although HTLV-1 PVL has been associated with the development and clinical progression of HAM/TSP [15]–[17], there was no significant difference between f-HAM/TSP and sporadic cases in the present study. Because previous studies reported that HTLV-1 PVLs of asymptomatic carriers in relatives of HAM/TSP patients were higher than those in non-HAM-related asymptomatic carriers [6], relatives of HAM/TSP are believed to be at a higher risk of developing HAM/TSP. Interestingly, our data suggest that HAM/TSP patients aggregate in families and factors other than HTLV-1 PVLs may contribute to HAM/TSP.

Compared with sporadic HAM/TSP, the clinical characteristics of f-HAM/TSP have a younger age of onset and longer time elapsed between onset and wheelchair use in daily life. Although we were unable to identify the reason for earlier onset among f-HAM/TSP cases, one can speculate that mild symptoms, such as urinary and sensory disturbances, may be identified earlier by family members who are familiar with HAM/TSP symptoms. However, the present data show no difference in initial symptoms between f-HAM/TSP and sporadic cases. In all cases, the age of onset and initial symptoms of HAM/TSP were evaluated by the neurologists during hospitalization. Because inflammatory processes are less marked in f-HAM/TSP cases, as indicated by significantly lower protein levels in CSF, f-HAM/TSP cases may show slow progression of disease.

We need to discuss the possibility that the two groups compared represent different mode of HTLV transmission, i.e. vertical vs. sexual transmission. To clarify genetic backgrounds, sporadic HAM/TSP with seropositive carrier family members may be a more appropriate control, but are not available at present. The incidence of female cases showing no significant differences between f-HAM/TSP and sporadic cases, and between rapid and slow disease progression, might suggest less possibility of sporadic cases due to sexual transmission.

Although the subgroup of patients with rapid progression has not been clearly defined, previous studies suggest that rapid progression occurs in 10%–30% of all patients with HAM/TSP [12], [14], [16], and is associated with an older age of onset [14]–[16]. In the present study, the age of onset in patients with rapid progression was significantly older than that in patients with slow progression between f-HAM/TSP and sporadic cases, and the proportion of patients with rapid progression increased with the older age of onset (Figure 2). Among sporadic cases, cell numbers and protein levels in CSF were significantly higher in patients with rapid progression, suggesting that inflammation is more active in the spinal cords of patients with rapid progression and that cytotoxic T-lymphocyte (CTL) immune responses may be more intensive. Therefore, lower PVLs in PBMCs of patients with rapid disease progression may be attributed to the strong killing ability of the CTL. However, PVLs were higher in PBMCs of patients with HAM/TSP than in asymptomatic carriers [6]. In addition, the killing ability of CTLs in patients with HAM/TSP does not differ from that in asymptomatic carriers [18]. Hence, strong immune responses may be associated with the disease course. The onset of disease may require other factors that lead to strong immune responses. A late onset may also be associated with alterations of the immune function in HTLV-1-infected patients. Indeed, an increased age has been associated with autoimmune disorders, such as myasthenia gravis and rheumatoid arthritis, and may be partly explained by immune intolerance and accumulation of autoantibodies in older individuals [19], [20].

In conclusion, we demonstrated that patients with HAM/TSP aggregate in some families. Compared with sporadic cases, the age of onset was younger and rates of disease progression were slower among familial cases, whereas HTLV-1 PVLs did not differ between f-HAM/TSP and sporadic groups. The present data suggest that factors other than HTLV-1 PVLs contribute to the disease course of HAM/TSP. Our data also suggested strong immune responses in the spinal cord of HAM/TSP patients with rapid progression. Further studies on HTLV-1, immune response to HTLV-1 and genetic factor in patients with rapid progression might provide new insights into HAM/TSP pathogenesis.
