# Correction: Familial Clusters of HTLV-1-Associated Myelopathy/Tropical Spastic Paraparesis

**DOI:** 10.1371/journal.pone.0152954

**Published:** 2016-03-30

**Authors:** Satoshi Nozuma, Eiji Matsuura, Toshio Matsuzaki, Osamu Watanabe, Ryuji Kubota, Shuji Izumo, Hiroshi Takashima

There is an error in the last sentence of the Results subsection of the Abstract. The correct sentence should read: HTLV-1 PVLs were lower in cases with rapid disease progression than in those with slow progression in sporadic cases.

A sentence is missing in the subsection “Clinical and laboratory findings in patients with rapid disease progression” of the Results section. The missing sentence should be added as the third to last sentence of that section and should read as: HTLV-1 PVLs of rapid progression were also lower than those of slow progression in familial cases (395 vs 984 copies), but it was not significant because of small number of subjects (3 rapid cases vs 29 slow cases).

There is an error in the subsection “Clinical characteristics of f-HAM/TSP” within the Results section. The ratio is flipped in the second sentence, which should correctly read: The sex ratio was 7 males: 33 females.

In Table 1, the values are incorrect for the “Female ratio (%)” row. Please see the corrected Table 1 here.

**Table 1:** Clinical features of f-HAM/TSP cases or sporadic cases of HAM/TSP

	f-HAM/TSP cases (40 cases)	Sporadic cases (124 cases)	p value	p value†
Female ratio (%)	82.5% (7 males: 33 females)	75.0% (31 males: 93 females)	NS	
Age	55.6 ± 13.0 (23–79)	61.8 ± 12.5 (15–83)	**0.008**	
Age of onset	41.3 ± 13.9 (14–65)	51.6 ± 15.9 (13–78)	**<0.001**	**0.017**
Duration of illness (years)	14.3 ± 11.4 (1–49)	10.2 ± 9.6 (0–45)	**0.026**	**0.017**
Initial symptoms				
Gait disturbance	50.0%	52.4%	NS	
Urinary disturbance	32.5%	26.6%	NS	
Sensory disturbance	12.5%	14.5%	NS	
Others	5%	6.5%	NS	
Rapid disease progression	4 cases (10.0%)	35 cases (28.2%)	**0.019**	0.069
Motor disability score	4.0 ± 2.0 (0–7)	4.9 ± 1.5 (0–8)	**0.043**	**0.036**
Score more than 6	12 cases (30.0%)	38 cases (30.7%)	NS	
Time elapsed between onset and wheelchair use in daily life (years)	18.3 ±12.4 (7–50)	10.0 ± 10.4 (1–45)	**0.025**	**0.020**

Data are presented as mean values ± s.d., (range)

**†** Adjusted for age and sex

The values are incorrectly reported in Fig 2. Please see the complete, correct Fig 2 here.

**Fig 2:**
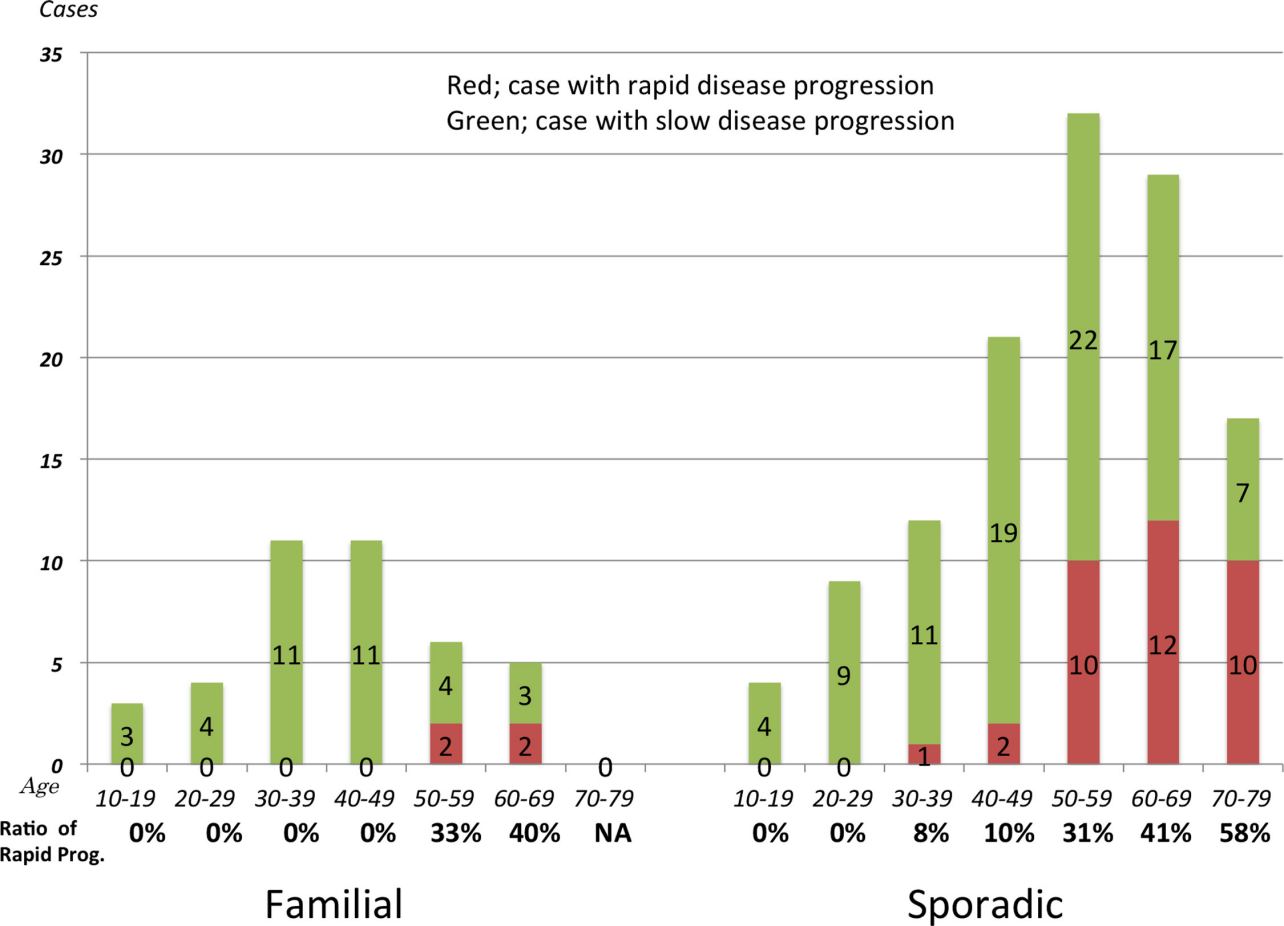
Age-specific proportions of rapid disease progression. The proportion of cases with rapid disease progression tended to increase with the older age of onset.
